# Dabrafenib and Trametinib prolong coagulation through the inhibition of tissue factor in BRAF^v600e^ mutated melanoma cells in vitro

**DOI:** 10.1186/s12935-019-0938-3

**Published:** 2019-08-28

**Authors:** Cristian Scatena, Sara Franceschi, Maria Franzini, Chiara Sanguinetti, Nadia Romiti, Laura Caponi, Mario Mandalà, Chiara Maria Mazzanti, Antonio Giuseppe Naccarato

**Affiliations:** 1 Division of Surgical Pathology, Department of Translational Research and New Technologies in Medicine and Surgery, University of Pisa, Via Roma 57, 56126 Pisa, Italy; 2 Fondazione Pisana per la Scienza, Pisa, Italy; 3 Division of Clinical Pathology, Department of Translational Research and New Technologies in Medicine and Surgery, University of Pisa, Pisa, Italy; 4 Unit of Medical Oncology, Department of Oncology and Haematology, Papa Giovanni XXIII Cancer Center Hospital, Bergamo, Italy

**Keywords:** Melanoma, Dabrafenib, Trametinib, Tissue factor, Coagulation, Raf/MEK/ERK pathway

## Abstract

**Background:**

Neoplastic cells promote a hypercoagulable state by the expression of cell surface proteins, such as tissue factor. In BRAF^v600^ mutated melanoma patients upon BRAF inhibitors, a hypercoagulable state correlates with prognosis, while a down-regulation of the hemostatic parameters is observed in patients responders as compared to non responders. The present study was intended to better clarify the strict relationship between coagulation mediators and target therapy in melanoma.

**Methods:**

The expression of tissue factor was investigated after the treatment with the BRAF inhibitor Dabrafenib and the MEK inhibitor Trametinib in the BRAF^v600e^ mutated melanoma cell lines A-375 and SK-MEL-28, together with its ability to activate the coagulation cascade.

**Results:**

Dabrafenib and Trametinib caused the down-regulation of TF in both cell lines A-375 and SK-MEL-28. For the cell line A-375 the effect was evident both at RNA and procoagulant activity; for the cell line SK-MEL-28 only at RNA level without any variation of the protein. Interestingly, when in contact with plasma deficient of factor VII, both cell lines were not able to activate the coagulation cascade.

**Conclusions:**

The present study provides the first in vitro observation that tissue factor expressed in melanoma cells may contribute to the modulation of the coagulation state of patients in the treatment with BRAF inhibitors.

## Background

Cancer patients commonly present abnormalities in coagulation markers and experience thromboembolic complications in 1–11% of cases [1]. Tumor cells have long been known to promote a hypercoagulable state by activating the hemostatic system through the expression of cell surface proteins, such as tissue factor (TF), cancer procoagulant (CP), tissue plasminogen activator (t-PA), urokinase plasminogen activator (uPA), and plasminogen activator inhibitor 1 (PAI-1) or 2 (PAI-2) [2]. Intriguingly, increasing evidence shows that key mediators of the hemostatic system represent essential players in cancer biology. The binding of activated factor VII (FVIIa) to TF expressed by tumor cells releases the negative regulatory control of protease-activated receptors (PARs), in particular PAR-1 and -2, resulting in the activation of several mitogen-activated protein kinase (MAPK) pathways, ultimately promoting angiogenesis, cell survival and metastasis [3]. In addition, thrombin promotes angiogenesis via clotting-dependent mechanisms—which involve platelets activation and fibrin deposition—and clotting-independent mechanisms mediated by TF-PARs signaling [4]. Melanoma cells express TF, thus contributing to metastatic dissemination through local generated proteolytic activity with the formation of a platelet thrombus, which determines the stable implantation of tumor cells in the microvasculature of target organs [5]. These data strongly suggest that both coagulation and melanoma progression are strictly linked. We recently reported on the thrombophilic status of BRAF^v600^ mutated melanoma patients upon BRAF inhibitors and showed that high d-dimer levels correlate with poor overall response rate, progression free survival and overall survival [6]. To clarify the relationship between coagulation mediators and the target therapy in melanoma, and in particular the modulation of the coagulation cascade in BRAF^v600^ mutated melanoma patients upon BRAF inhibitors, we herein investigated the effects of BRAF and MEK inhibitors, alone or in association, on TF expression in BRAF^v600e^ mutated melanoma cell lines. Moreover, TF procoagulant activity was evaluated in plasma in order to better understand the role of TF expressed by melanoma cells in the coagulation cascade.

## Methods

### Chemicals

The BRAF inhibitor Dabrafenib (GSK2118436) and the MEK1 and MEK2 inhibitor Trametinib (GSK1120212) were purchased by Selleck Chemicals (http://www.selleckchem.com).

### Cell lines

The human melanoma cell lines A-375 and SK-MEL-28, carrying BRAF^v600e^ mutation, were obtained from the American Type Culture Collection (ATCC, Manassas VA). The cultures were grown in Dulbecco’s Modified Eagle’s Medium (DMEM) containing 4 mM l-glutamine; 4500 mg/L glucose; 1 mM sodium pyruvate and 1500 mg/L sodium bicarbonate, in the presence of 10% FBS; and 1% penicillin–streptomycin. To ensure the quality and integrity of human cell lines, cells from the initial thawed vials were used for up to a maximum of 10 passages in all the experiments, as recommended by the supplier. Cells were tested for the presence of mycoplasma (EZ-PCR Mycoplasma Test Kit; Biological Industries, Beth Haemek, Israel) with negative results.

### Cell growth inhibition assay

The half maximal inhibitory concentration (IC_50_) was estimated in A-375 and SK-MEL-28 cells after 3-days treatment with increasing concentrations of Dabrafenib and Trametinib (from 0 nM to 10 μM), using WST-1 premixed Cell Proliferation Assay Kit (Clontech) according to the manufacturer’s protocol. The quantity of formazan dye is directly related to the number of metabolically active cells and was quantified by measuring the absorbance at 450 nm in a multiwell plate reader (Tecan, Mannedorf, Switzerland). OD values after 3 days were normalized to T0 and cell growth was calculated as relative % of control (DMSO). Experiments were performed as technical triplicates over biological triplicates, with a total of 9 data points. Data were presented as mean ± SD and differences were considered statistically significant when p < 0.05. IC_50_ was estimated as 1 nM for Dabrafenib and 0.1 nM for Trametinib in A-375 cells and 10 nM for Dabrafenib and 1 nM for Trametinib in SK-MEL-28 cells (Additional file 1).

### RNA extraction

RNA was extracted in triplicate from A-375 and SK-MEL-28 cells cultured in a 96 well plate after 48 h the addition of Dabrafenib and Trametinib alone and the combination of the two compounds (IC_50_ of 1 nM Dabrafenib and 0.1 nM Trametinib in A-375, 10 nM Dabrafenib and 1 nM Trametinib in SK-MEL-28) or the vehicle dimethyl sulfoxide (DMSO) alone. Two hundred and twenty-five microliter of Lysis Buffer were added and scratches were performed using a 100 µL tip after the cell medium was removed. Twenty-five microliter of proteinase K were added and the extraction was continued according to the Maxwell 16 LEV RNA FFPE Kit (Promega) protocol. The RNA concentration was measured using a Qubit 2.0 Fluorometer (Life Technologies). The RNA yield ranged from 50 to 500 ng/µL.

### Gene expression analysis

For each sample 5 µL (100 ng) of RNA were reverse transcribed using the RT-NanoScript kit (PrimerDesign) in a final volume of 20 µL containing 10× RT nanoScript buffer; 10 mM dNTPs; Random Nonamer primer; 100 mM DTT, RNase/DNase free water; and the nanoScript enzyme. Real time PCR (Biorad CFX96). For each sample real time PCR was performed in a final volume of 20 µL containing 1× SsoAdvanced SYBR Green Supermix (Biorad), 400 nM of primers (Table 1) and 2 µL of cDNA. The amplification profile was as follows: 95 °C for 2 min; 40 cycles at 95 °C for 5 s; and 60 °C for 30 s. GAPDH CT (cycle threshold) values were used for normalization. Experiments were performed as technical triplicates over biological triplicates, with a total of 9 data points. Data were presented as mean ± SD and differences were considered statistically significant when p < 0.05.

**Table 1:** Forward (F) and reverse (R) primer sequences for real time PCR

TF_F	TGTATGGGCCAGGAGAAAGG
TF_R	CCCACTCCTGCCTTTCTACA
GAPDH_F	ATTGCCCTCAACGACCACTT
GAPDH_R	TGCTGTAGCCAAATTCGTTGT

### Protein extraction

Proteins were extracted from A-375 and SK-MEL-28 cells cultured in 6 well plates after 48 h (T1) the addition of both compounds (IC_50_ of 1 nM and 0.1 nM for Dabrafenib and Trametinib, respectively), alone and in combination, or the vehicle DMSO alone. Each cell pellet was treated with 50 µL RIPA buffer (Sigma) and 1.5 µL Protease/Phosphatase Inhibitor Cocktail (Cell Signaling Technology), incubated on ice for 30 min, with 5 s of vortex every 5 min, and then centrifuged (14,000 RPM for 30 min at 4 °C). The supernatant (total protein) was quantified with Qubit 2.0 Fluorometer (Life Technologies) using 2 µL of 1:100 diluted protein solution. The total protein yield ranged from 15 to 30 µg/µL.

### Western blot analysis

For each sample, 40 μg of proteins were loaded on the 10% Mini-PROTEAN TGX Gel (Bio-Rad). Proteins were transferred from gels to membrane with the Trans-Blot Turbo transfer system (Bio-Rad). The concentration of the primary antibodies are listed in Table 2. Protein detection was performed using the Bio-Rad Clarity western ECL substrate (Bio-Rad). Secondary antibodies Goat Anti-Mouse IgG H&L (HRP) (ab6789, Abcam) and Goat Anti-Rabbit IgG H&L (HRP) (ab6721, Abcam) were used at a dilution of 1:2000. The ChemiDoc MP imager was used to detect the chemiluminescent signal. Densitometry was performed using the open-source software ImageJ [7]. The abundance of the TF protein was normalized to the total amount of the housekeeping protein (β-tubulin) in each lane, and relative expressions were calculated in comparison to the control (DMSO) (Additional file 3).

**Table 2:** Genes and primary antibodies used for Western blot analysis

Gene	Antibody	Dilution	Host
TF	WH0002152M1 (Sigma Aldrich)	1:100	Mouse
β-tubulin	sc-9104 (Santa Cruz)	1:1000	Rabbit

### Immunocytochemistry

A-375 and SK-MEL-28 cell lines were plated on glass slides as a monolayer and processed after 48 h (T1) the addition of both compounds (IC_50_ of 1 nM and 0.1 nM for Dabrafenib and Trametinib, respectively), alone and in combination, or the vehicle DMSO alone. The slides were fixed with ethanol for 5 min, and then washed three times in PBS to remove fixative. Slides were dipped in PBS Tween 20 (0,1% v/v) for 10 min to permeabilize cell membranes and washed three times in PBS. Slides were put in the moist chamber. Endogenous peroxidase was blocked by 3% hydrogen peroxide for 15 min. The antigen unmasking was achieved with MS-unmasker solution (DIAPATH, Martinengo, BG, Italy) in microwave. We used Mouse specific HRP/DAB (ABC) Detection IHC Kit (ab64259) (Abcam, Cambridge, UK) according to the manufacturer’s protocol. The slides were incubated with the primary antibodies and TF monoclonal antibody WH0002152M1 (Sigma Aldrich, St. Louis, MO, USA) at a dilution of 1:500, 1 h at room temperature. Slides were then incubated for 30 min with biotinylated goat antimouse IgG secondary antibody provided in the kit. ABC avidin/biotin reagent was added to the slides and incubated for 10 min. Slides were developed with diaminobenzidine chromogen (DAB) and counterstained with hematoxylin. They were analyzed using an inverted microscope CARL ZEISS “Axio Observer Z1FLMot”, and images were taken with CARL ZEISS “AXIOCAM Icc1” camera.

### Tissue factor procoagulant activity

Tissue factor activity was measured by a clotting assay performed on a monodisperse cell suspension obtained by trypsinization. In details, test was performed in a 96-well microplate mixing 100 µL of cell suspension containing 300,000 cells and 100 µL of normal pool plasma (NPP); after 3 min at 37 °C, 50 µL CaCl_2_ were added by a multichannel pipette to trigger coagulation. The reaction was followed measuring the variation of absorbance at 671 nm for 600 s (200 readings every 3 s) at 37 °C (Multiskan GO, Thermo Fisher). The test was performed in quadruple and the blank of the reaction was obtained using 100 μL of Dulbecco’s phosphate buffered saline instead of cell suspension. The time required for the formation of the first fibrin filaments (clotting time) was measured as reported in Additional file 2. Clotting times were converted into Unit of TF activity on the base of a calibration curve obtained from a scalar dilution of the RecombiPlasTin 2G (Werfen), a high sensitivity thromboplastin reagent based on recombinant human tissue factor. 1U of TF activity was defined as the quantity of TF able to trigger coagulation in 50 s. Cell suspensions were also analyzed using 100 μL of factor VII or factor XII deficient plasma (Werfen HemosIL) in place of NPP. NPP was obtained by mixing sodium citrate plasma aliquots obtained from 20 healthy volunteers. The factor VII and XII activities of the NPP were 104.5% and 91.7%, respectively. Cell suspensions were also analyzed using 100 μL of FVII or FXII deficient plasma (Werfen HemosIL) in place of NPP. These two coagulation factors represent the two possible activators of coagulation in vitro: FVII can activate coagulation only when bound to TF and viceversa, FXII instead is activated by negative surface and its coagulative pathway is independent of the activity of the complex TF/FVII. Since TF in absence of FVII can not be activated, data of the experiments with factor deficient plasma were reported as clotting time (s). All experiments were performed three times, and within each experiments data were collected in triple or quadruple. Data are reported as mean ± standard deviation.

### Statistical analysis

The results of the expression levels of TF and its procoagulant activity in A-375 and SK-MEL-28 cells were statistically analyzed by unpaired Student’s t-test. Data sets derived from WST-1 analysis were screened by one-way analysis of variance (ANOVA).

## Results

### Dabrafenib and Trametinib decrease TF expression in BRAF^v600e^ melanoma cell lines

**Fig. 1:**
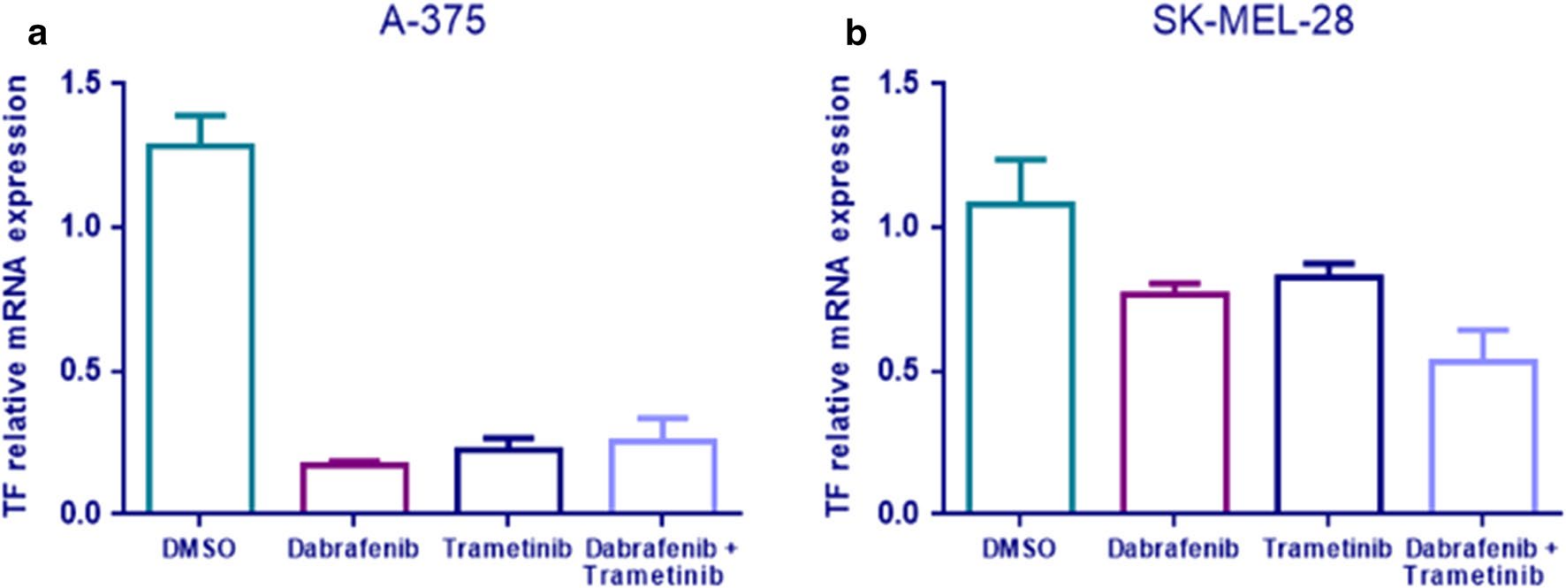
TF mRNA reduction after Dabrafenib and Trametinib treatment. RT-PCR quantification of A375 (**a**) and SK-MEL-28 (**b**) cells treated with Trametinib/Dabrafenib for 48 h. Data are expressed as the mean ± standard deviation. Experiments were performed as technical triplicates over biological triplicates, with a total of 9 data points. Data were presented as mean ± SD and differences were considered statistically significant when p < 0.05

**Fig. 2:**
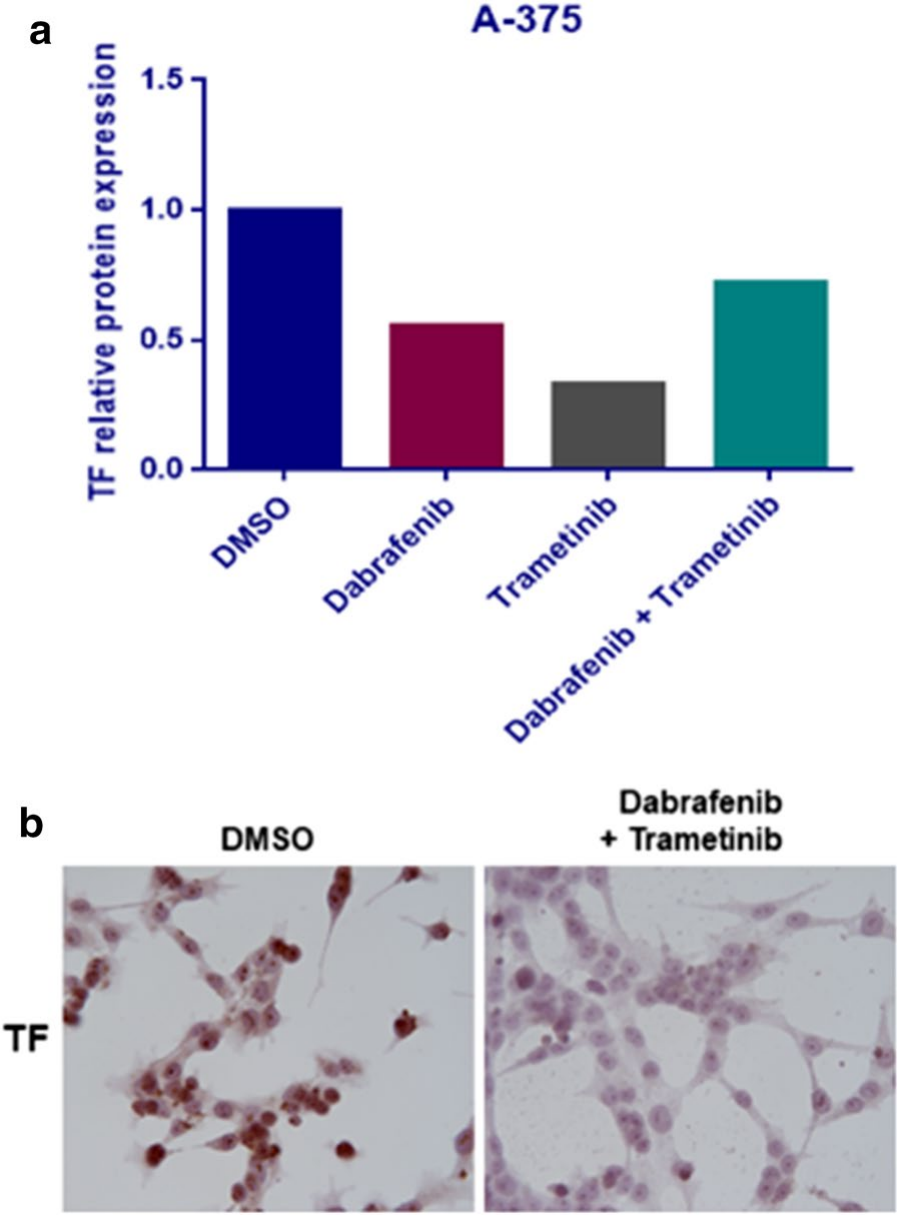
TF protein reduction in A-375 cells treated for 48 h with the combination Dabrafenib and Trametinib compared to DMSO as control. The istograms describe the reduction by Western blot analysis (**a**), confirmed by immunocytochemistry (**b**)

**Fig. 3:**
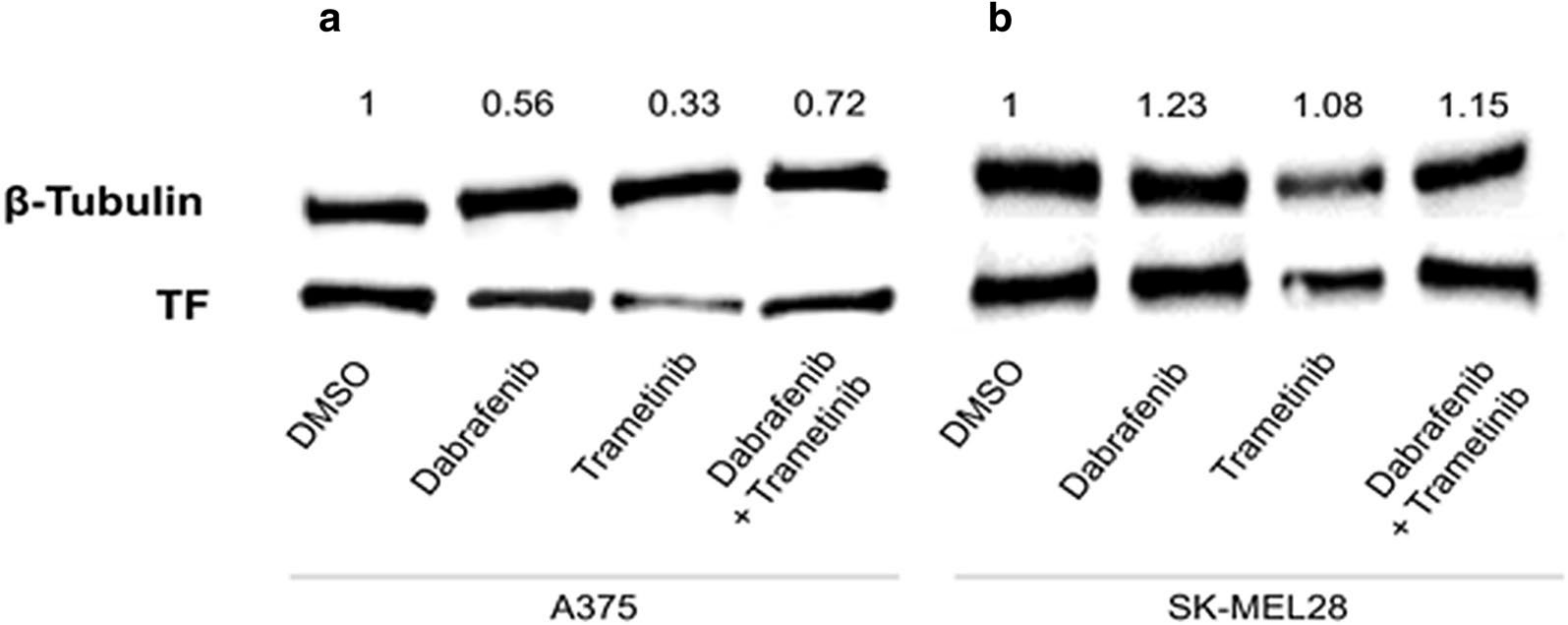
Western blot analysis of total protein extracts of A-375 (**a**) and SK-MEL-28 (**b**) cells treated for 48 h with Dabrafenib and Trametinib, alone and in combination, and DMSO as control. The protein species were detected at the expected molecular weights per their datasheets, as follows: β-tubulin ~ 55 kDa; tissue factor (TF) ~ 35 kDa. Protein expression was normalized to their loading control (β-tubulin)

Dabrafenib and Trametinib caused the decrease of TF mRNA expression in both A-375 and SK-MEL-28 cell lines. In A-375 cell line there is a significant 7.4-fold reduction of TF mRNA in Dabrafenib treated cells (p < 0.0001); 5.6-fold reduction in Trametinib treated cells (p < 0.0001); and 5.1-fold reduction in cells treated with the combination of Dabrafenib + Trametinib (p = 0.0002) (Fig. 1a). SK-MEL-28 treatment with Dabrafenib and Trametinib causes a significant decrease in TF mRNA of approximately 1.5-fold (p = 0.0255 Dabrafenib, p = 0.0498 Trametinib and p = 0.0074 Dabrafenib + Trametinib) (Fig. 1b). Moreover, in A-375 is also evident a TF protein expression reduction after Dabrafenib and Trametinib treatment (Figs. 2, 3a), whereas in SK-MEL-28 there is not a significant reduction in TF protein expression (Fig. 3b and Additional file 3).

### Dabrafenib and Trametinib decrease TF activity of BRAF^v600e^ melanoma cell lines

**Fig. 4:**
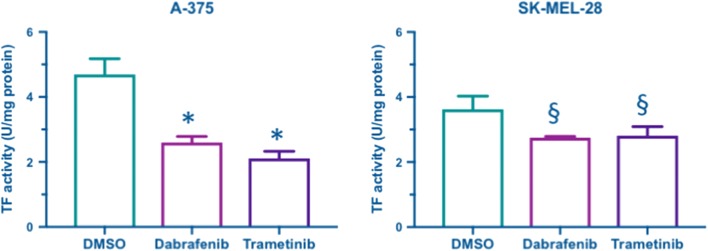
Tissue factor activity in A-375 and SK-MEL-28 cells after 48 h of treatment with Dabrafenib, Trametinib or DMSO as control. TF activity is expressed as U/mg of protein and it has been measured by a clotting assay conducted on monodisperse cell suspension as described in “Methods” section. Data are from a representative experiment of three and are reported as mean ± standard deviation of four replicates. Statistical analysis: 1 way-ANOVA and Tukey’s multiple comparisons test: ^§^p < 0.01, *p < 0.0001 vs. DMSO

**Fig. 5:**
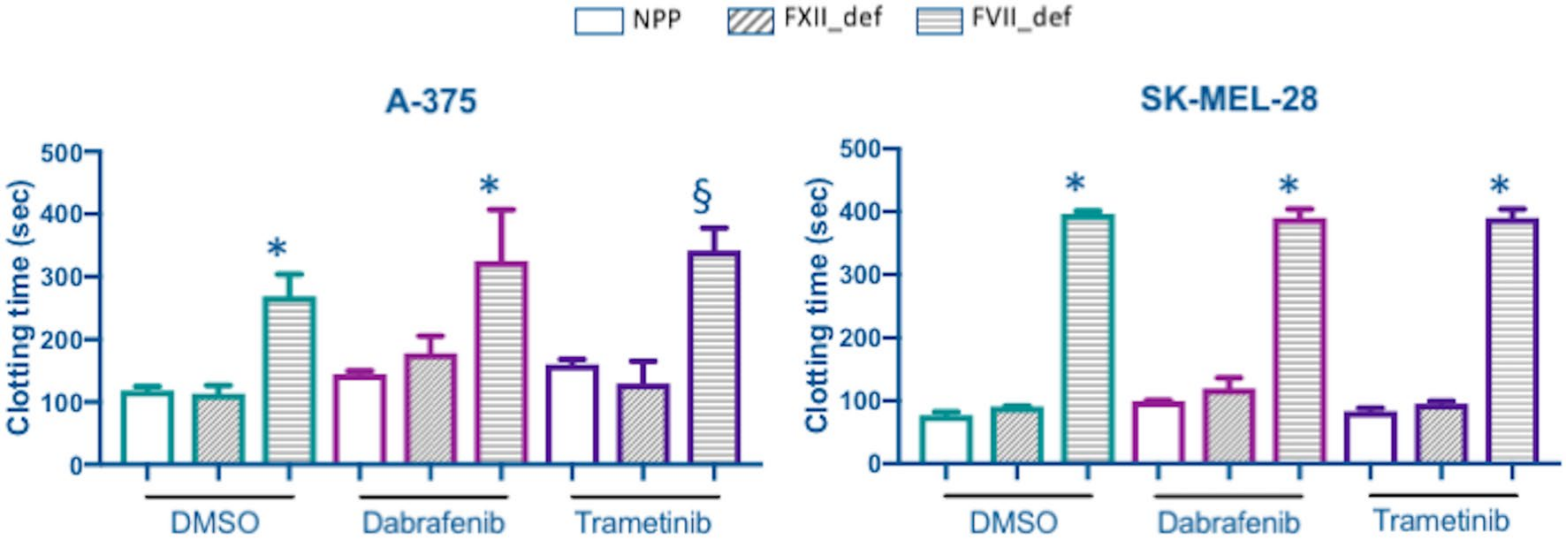
Clotting time (s) measured in A-375 and SK-MEL-28 cells, after 48 h of treatment with Dabrafenib, Trametinib or DMSO as control, in presence of normal pool plasma (NPP) and in factor XII (FXII_def) or VII (FVII_def) deficient plasma. Clotting assays were conducted on monodisperse cell suspension as described in “Methods” section. Data are from a representative experiment of three and are reported as mean ± standard deviation of four replicates for NPP and three replicates for factor deficient plasma. Statistical analysis: 1 way-ANOVA and Tukey’s multiple comparisons test: ^§^p < 0.01, *p < 0.0001 vs. NPP

TF activity was measured in both A-375 and SK-MEL-28 cells after 48 h of incubation with DMSO, Dabrafenib or Trametinib. The analysis was conducted on monodisperse cell suspension to measure TF activity in its native phospholipid context; to evaluate the specific contribution of TF in activating coagulation, clotting time was also measured in NPP and in factor XII (FXII_def) or VII (FVII_def) deficient plasma. Both Dabrafenib and Trametinib reduced significantly TF activity compared to controls in DMSO in both cell models studied (Fig. 4). The comparison among clotting times observed in NPP and factor deficient plasma confirmed that TF-mediated activation of coagulation was the prevalent one regardless of treatment: in FVII deficient plasma, where TF can not be activated, clotting time was prolonged, while in FXII deficient plasma clotting time was comparable to that in NPP (Fig. 5).

## Discussion

For the first time to our best knowledge, we demonstrated that the BRAF inhibitor Dabrafenib and the MEK inhibitor Trametinib [8] down-regulate TF in BRAF^v600e^ mutated melanoma cells with a consequent inhibition of the coagulation cascade. TF is a transmembrane glycoprotein and represents the primary initiator of normal blood coagulation; its expression characterizes many malignant tumours, melanoma included [5, 9–11] where it correlates with a hypercoagulation status [6]. Our findings are in line with the lately reported clinical observations. Indeed, Verzeroli et al. [12] observed a down-regulation of the hemostatic parameters thrombin generation potential of plasma (TG) and d-dimer in patients who responded > 85% to BRAF^v600e^ inhibitor therapy as compared to patients who responded < 85% or did not respond. The authors concluded that this observation pointed to a possible association between hypercoagulation, BRAF^v600e^ mutation and response to therapy. Therefore, we may suppose that the down-regulation of TF in melanoma cells could be responsible of the decrease levels of TG and d-dimer observed in patients with an efficient therapeutic response to BRAF inhibitors; on the contrary, in patients who respond < 85% or do not respond high levels of coagulation markers could be maintained since BRAF inhibitors may also fail to down-regulate TF in melanoma cells. The effect on TF was evident for the cell line A-375, both at RNA and protein levels; on the contrary, the SK-MEL-28 cell line displayed a significant inhibition of TF expression at RNA level without any variation of the protein. Several hypotheses can be formulated to explain this discrepancy. As showed in Fig. 3, a significant decreases of TF expression was observed at mRNA level, with however differences between the two cell lines tested: a one- to twofold decrease for SK-MEL-28 and a five- to sevenfold decrease for A375 cells. According to this, Western blot represents an effective early analytical tool to identify a protein of interest in a complex mixture however, as with all techniques, it has its limitations: several commercial antibodies may indeed exhibit off-target effects by interacting with other proteins [13]. However, even if non-specific interactions are minimized by lowering the concentration of the primary antibody and/or varying the period of incubation time, they may mask small decrease in the target protein. A second possible explanation may be in the incorrect choice of the temporal window: SK-MEL-28 may in fact require more time to decrease its TF protein content. Furthermore, even if the role of the various domains of the primary structure of membrane-bound TF on its procoagulant activity are well established, little is known about the influence of post-translational modification on its function. Interestingly, only a small fraction of membrane-bound TF supports the coagulation whereas most of the TF remains unfunctional, known as the cryptic TF; thus, protein expression and procoagulant activity may not match [14–16]. The mechanisms of the conversion from cryptic TF to active TF (decryption) and viceversa (encryption) are numerous and complex: (1) increased availability of anionic phospholipids at the cell surface is responsible for the conversion from cryptic TF to procoagulant TF [17]; (2) cryptic form of TF exists as dimers on cell surface where it is converted into procoagulant TF after a process of monomerization triggered by calcium influx into cytosol [18]; (3) distribution of TF on cell surface is not random: evidence support the hypothesis that the criptic form of TF is associated with caveolae, a specialized membrane domain rich in cholesterol and sphingolipid rafts [19].

Nevertheless, the inhibition of TF was confirmed by the functional assay where for both cell lines the treatment with Dabrafenib or Trametinib inhibited the coagulation cascade.

According to this complex scenario, we may speculate that BRAF and MEK inhibitors decrease TF activity in both cell lines tested through two different processes. In A375 cells Dabrafenib and Trametinib may decrease TF procoagulant activity by the decrease of the amount of TF protein, since both mRNA and protein (by WB analysis and immunocytochemistry) decrease significantly. The precise mechanisms through which BRAF and MEK inhibitors may down-regulate TF in melanoma cells are at present unknown. BRAF inhibition with Dabrafenib and MEK inhibition with Trametinib, either as monotherapy or in combination, lead to the suppression of phosphorylated ERK (pERK) in melanoma cells [20]. The ERK cascade is involved in many signaling transduction pathways [21], and in melanoma, its inhibition affects the tumour microenvironment, with impact on T cells, dendritic cells, tumor cells, stromal cells and soluble factors [22, 23]. Interestingly, in recent years the ERK cascade has been associated with the regulation of coagulation, with particular attention to TF. Chang et al. [24] first reported that activated MEK/ERK pathways specifically phosphorylate and transactivate transcription factors such as Elk-1, which then induce the expression of TF. In particular, this mechanism has been reported to explain the increased expression of TF on the surface of monocytes [25] and endothelial cells (ECs) [26] in patients with antiphospholipid syndrome. Thus, the blockade of the MEK-1/ERK pathway could induce the inhibition of monocyte and EC TF expression and this mechanism has represented an attractive avenue for the development of treatments to prevent thrombosis. The same mechanism has been described in the activation of TF expression in breast and colorectal carcinoma cells [27, 28]. Therefore, according to literature, the inhibition of TF in A375 melanoma cells by the selective blockade of the Raf/MEK/ERK pathway may be explained with the inhibition of transactivation of specific transcription factors that constitutively induce the expression of TF. On the other hand, in SK-MEL-28 cells a process of encryption of TF protein may decrease its procoagulant activity keeping its amount unchanged.

The two different processes at the base of the reduction of TF activity in A375 and SK-MEL-28 melanoma cells may be reasonable since even if both cell lines derive from human metastatic cutaneous melanoma cells bearing V600E mutations in BRAF gene, they differ in proliferation, migration and invasion rates, with the A375 showing a more aggressive phenotype compared to SK-MEL-28 [29]. Thus, we may speculate that the two cell lines tested may also differ in the mechanisms through which they modulate membrane-bound TF procoagulant activity. In order to confirm these hypotheses, in vitro experiments are needed. However this overcomes the aim of the present study that pointed to verify the effect of the two drugs on TF activity.

## Conclusions

Our data provide the first in vitro observation that BRAF/MEK inhibitors decrease TF in BRAF^v600e^ mutated melanoma cells thus inhibiting the coagulation cascade, adding one more piece of evidence to the complex relationship between coagulation and cancer.

## Supplementary information

**Additional file 1.** Cell growth inhibition assay. BRAF^v600e^ melanoma cell lines sensitivity to Dabrafenib and Trametinib.

**Additional file 2.** Clotting time calculation. Calculation of the time required for the formation of the first fibrin filaments (clotting time).

**Additional file 3.** Biological repeats of WB analysis for SK-MEL-28 cell line. Repetition of Western blot analysis on samples collected in the three experiments for TF activity evaluation from the SK-MEL-28 cell line.

## Data Availability

All data generated or analysed during this study are included in this published article (and its additional files)

## Abbreviations

TF: tissue factor
CP: cancer procoagulant
t-PA: tissue plasminogen activator
uPA: urokinase plasminogen activator
PAI-1: plasminogen activator inhibitor 1
PAI-2: plasminogen activator inhibitor 2
FVIIa: activated factor VII
MAPK: mitogen-activated protein kinase
DMEM: Dulbecco’s Modified Eagle’s Medium
IC_50_: the half maximal inhibitory concentration
DMSO: dimethyl sulfoxide
CT: cycle threshold
DAB: diaminobenzidine chromogen
NPP: normal pool plasma
TG: thrombin generation potential of plasma
pERK: phosphorylated ERK
