# Supplementary material for: Dabrafenib and Trametinib prolong coagulation through the inhibition of tissue factor in BRAFv600e mutated melanoma cells in vitro
Source: Cancer Cell Int. 2019 Aug 28;19:223. doi: 10.1186/s12935-019-0938-3 (PMC6712666; doi:10.1186/s12935-019-0938-3)
Supplement: Supplementary file 1 — Additional file 1. Cell growth inhibition assay. BRAFv600e melanoma cell lines sensitivity to Dabrafenib and Trametinib. [file 12935_2019_938_MOESM1_ESM.pdf]

Cell growth inhibition assay

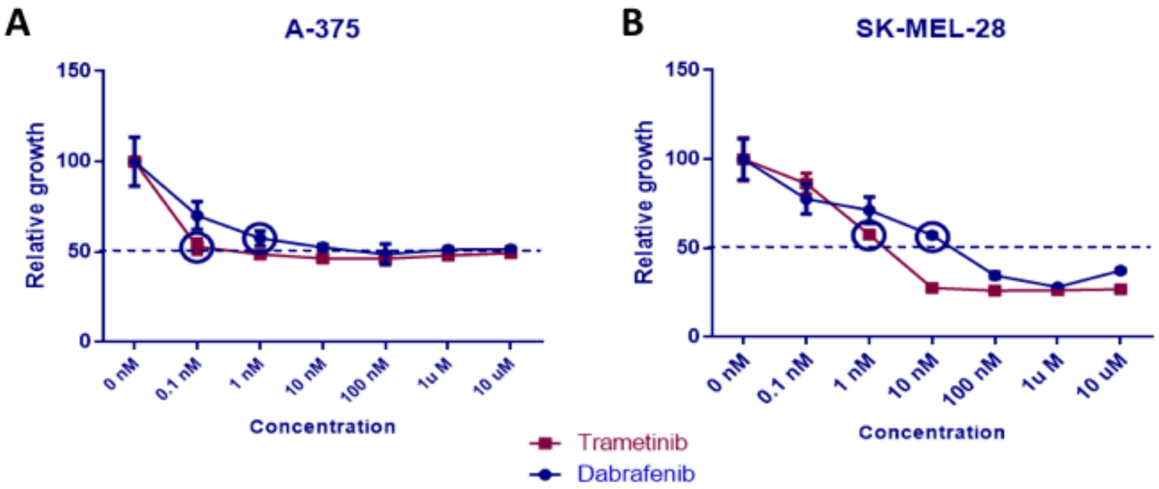

BRAF^{v600e} melanoma cell lines sensitivity to Dabrafenib and Trametinib. A375 (A) and SK-MEL-28 (B) cells were treated with various concentrations of Dabrafenib and Trametinib for 72 hours. Following the treatment, cell viability was evaluated with WST1 test and IC50 was estimated as the minimum concentration required to reduce cell growth to 50% of the control value (0 nM). Experiments were performed as technical triplicates over biological triplicates, with a total of 9 data points. Data were presented as mean ± SD and differences were considered statistically significant when $p < 0.05$.
