# Supplementary material for: Dabrafenib and Trametinib prolong coagulation through the inhibition of tissue factor in BRAFv600e mutated melanoma cells in vitro
Source: Cancer Cell Int. 2019 Aug 28;19:223. doi: 10.1186/s12935-019-0938-3 (PMC6712666; doi:10.1186/s12935-019-0938-3)
Supplement: Supplementary file 2 — Additional file 2. Clotting time calculation. Calculation of the time required for the formation of the first fibrin filaments (clotting time). [file 12935_2019_938_MOESM2_ESM.pdf]

Clotting time calculation

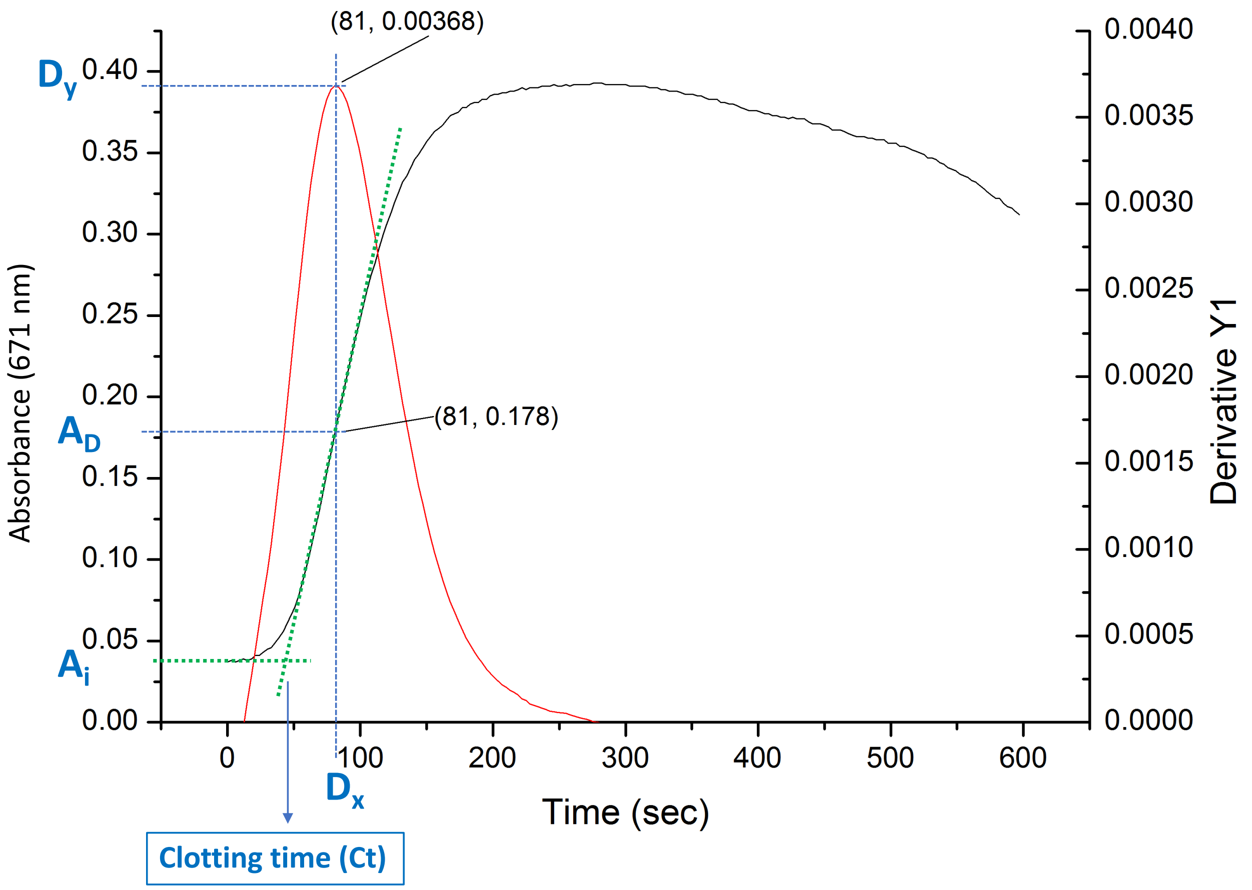

The peak of the first derivative (Dx, Dy) locates the point of maximum speed of the reaction. The value (Dy) of the first derivative in Dx correspond to the slope of the tangent line at the point (D_x, A_D)

The equation of the tangent line and passing through (D_x, A_D) follows:

$$\begin{cases} y = mx + b \\ m = D_y \\ b = A_D - D_y D_x \end{cases}$$

$$y = D_y x + A_D - D_y D_x$$

The coordinate (Ct) of the intersection point is:

$$\begin{cases} y = D_y x + A_D - D_y D_x \\ y = A_i \end{cases}$$

$$x = Ct = \frac{A_i - A_D + D_y D_x}{D_y}$$
