# Supplementary material for: Dabrafenib and Trametinib prolong coagulation through the inhibition of tissue factor in BRAFv600e mutated melanoma cells in vitro
Source: Cancer Cell Int. 2019 Aug 28;19:223. doi: 10.1186/s12935-019-0938-3 (PMC6712666; doi:10.1186/s12935-019-0938-3)
Supplement: Supplementary file 3 — Additional file 3. Biological repeats of WB analysis for SK-MEL-28 cell line. Repetition of Western blot analysis on samples collected in the three experiments for TF activity evaluation from the SK-MEL-28 cell line. [file 12935_2019_938_MOESM3_ESM.pdf]

Biological repeats of WB analysis for SK-MEL-28 cell line

Western blot analysis was repeated on samples collected in the three experiments for TF activity evaluation from the SK-MEL-28 cell line.

Protein detection was performed using the Roche ECL substrate instead of Bio-Rad Clarity western ECL substrate which was not available; the ChemiDoc MP imager auto-expose tool was used to detect the chemiluminescent signal. The abundance of the TF protein was normalized to the total amount of the housekeeping protein (β-tubulin) in each lane; densitometry was performed using the open-source software ImageJ.

The images for densitometry follow:

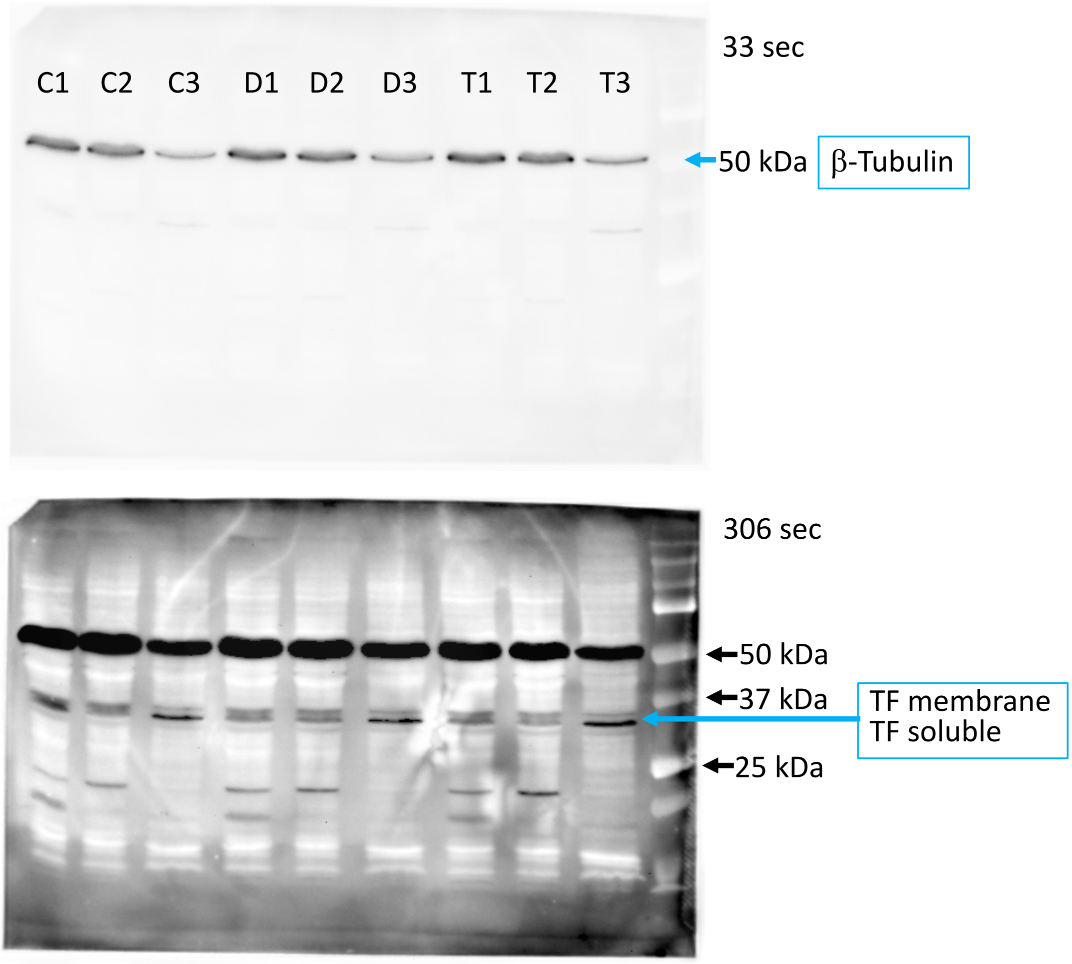

With the Roche ECL substrate two bands were detectable at the molecular weight of TF likely corresponding to the membrane (263 aa, ~35 kDa) and soluble isoforms of TF (206 aa, ~30 kDa).

Analysis of western blot confirmed no changes in both TF isoforms expression in the three examined experiments.

	Band density – Chemiluminescence								
IntDen	**C1**	**C2**	**C3**	**D1**	**D2**	**D3**	**T1**	**T2**	**T3**
β-Tubulin	460.94	439.19	206.53	490.11	445.79	265.26	512.23	452.96	256.18
TF membrane	202.89	253.29	182.44	215.14	254.59	228.71	207.96	197.05	247.34
TF soluble	282.10	233.74	342.49	227.31	256.66	362.61	260.76	196.14	504.59
ratio TFm/tub	0.440	0.577	0.883	0.439	0.571	0.862	0.406	0.435	0.966
ratio TFs/tub	0.612	0.532	1.658	0.464	0.576	1.367	0.509	0.433	1.970
ratio TFm+TFs/tub	1.052	1.109	2.542	0.903	1.147	2.229	0.915	0.868	2.935
	mean	*SD*		*mean*	*SD*		*mean*	*SD*	
TFm/tub	0.633	0.227		0.624	0.217		0.602	0.315	
TFs/tub	0.934	0.628		0.802	0.492		0.971	0.866	
TFm+TFs/tub	1.568	0.844		1.426	0.706		1.573	1.180	
				Test T paired data: D vs. C			*Test T paired data: T vs. C*		
TFm/tub				0.263			0.677		
TFs/tub				0.307			0.816		
TFm+TFs/tub				0.297			0.981		
							Test T paired data: T vs.D		
TFm/tub							0.782		
TFs/tub							0.530		
TFm+TFs/tub							0.666		

Since the background in the chemiluminescence detection was quite strong, the same membrane was washed with PBS and developed again with the colorimetric Opti-4CN substrate (Bio-Rad); the membrane image was acquired with an Epson scanner. Densitometric analysis was repeated with similar results.

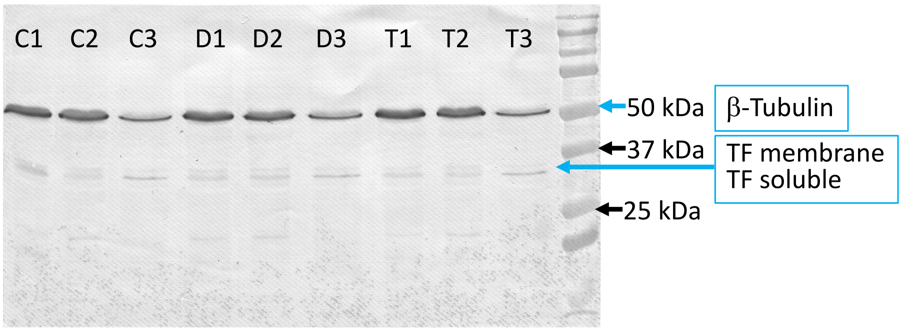

IntDen	C1	C2	C3	D1	D2	D3	T1	T2	T3
	**Band density - Colorimetric**								
β-Tubulin	919.95	979.65	1192.70	965.45	1004.52	1159.46	963.11	995.02	1193.16
TF membrane	544.63	555.54	565.71	557.89	558.59	563.18	570.31	562.11	561.59
TF soluble	526.22	546.64	534.75	543.43	540.91	534.28	551.36	553.62	532.20
TFm/tub	0.592	0.567	0.474	0.578	0.556	0.486	0.592	0.565	0.471
TFs/tub	0.572	0.558	0.448	0.563	0.538	0.461	0.572	0.556	0.446
TFm+TFs/tub	1.164	1.125	0.923	1.141	1.095	0.947	1.165	1.121	0.917
	mean	*SD*		mean	SD		*mean*	*SD*	
TFm/tub	0.544	0.062		0.540	0.048		0.543	0.064	
TFs/tub	0.526	0.068		0.521	0.053		0.525	0.069	
TFm+TFs/tub	1.071	0.130		1.061	0.101		1.068	0.132	
				Test T paired data: D vs. C			*Test T paired data: T vs. C*		
TFm/tub				0.627			0.227		
TFs/tub				0.624			0.303		
TFm+TFs/tub				0.617			0.255		
							Test T paired data: T vs. D		
TFm/tub							0.793		
TFs/tub							0.707		
TFm+TFs/tub							0.742		
